# Interactions of the Brain Renin-Angiotensin-System (RAS) and Inflammation in the Sensitization of Hypertension

**DOI:** 10.3389/fnins.2020.00650

**Published:** 2020-07-15

**Authors:** Baojian Xue, Yuping Zhang, Alan Kim Johnson

**Affiliations:** ^1^Department of Psychological and Brain Sciences, The University of Iowa, Iowa City, IA, United States; ^2^Department of Pathophysiology, Hebei North University, Zhangjiakou, China; ^3^Neuroscience and Pharmacology, The University of Iowa, Iowa City, IA, United States; ^4^Health and Human Physiology, The University of Iowa, Iowa City, IA, United States; ^5^The François M. Abboud Cardiovascular Research Center, The University of Iowa, Iowa City, IA, United States

**Keywords:** RAS, inflammation, CNS, hypertension, perinatal programming

## Abstract

Mounting evidence indicates that the renin-angiotensin (RAS) and immune systems interact with one another in the central nervous system (CNS) and that they are importantly involved in the pathogenesis of hypertension. Components comprising the classic RAS were first identified in the periphery, and subsequently, similar factors were found to be generated *de novo* in many different organs including the brain. There is humoral-neural coupling between the systemic and brain RASs, which is important for controlling sympathetic tone and the release of endocrine factors that collectively determine blood pressure (BP). Similar to the interactions between the systemic and brain RASs is the communication between the peripheral and brain immune systems. Systemic inflammation activates the brain’s immune response. Importantly, the RAS and inflammatory factors act synergistically in brain regions involved in the regulation of BP. This review presents evidence of how such interactions between the brain RAS and central immune mechanisms contribute to the pathogenesis of hypertension. Emphasis focuses on the role of these interactions to induce neuroplastic changes in a central neural network resulting in hypertensive response sensitization (HTRS). Neuroplasticity and HTRS can be induced by challenges (stressors) presented earlier in life such as a low-dose of angiotensin II or high fat diet (HFD) feeding in adults. Similarly, the offspring of mothers with gestational hypertension or of mothers ingesting a HFD during pregnancy are reprogrammed and manifest HTRS when exposed to new stressors as adults. Consideration of the actions and interactions of the brain RAS and inflammatory mediators in the context of the induction and expression of HTRS will provide insights into the etiology of high BP that may lead to new strategies for the prevention and treatment of hypertension.

## Introduction

Hypertension is a major risk factor for the development of cardiovascular diseases that are responsible for high morbidity and mortality. Approximately one-third of adults in the United States have hypertension, and only half of hypertensive patients show a satisfactory response to treatment (Go et al., 2014; Fryar et al., 2017). This may be because many of the existing antihypertensive therapies target peripheral mechanisms and are not the most effective for treating those forms of hypertension that are driven from the central nervous system (CNS) (Go et al., 2014). A better understanding of CNS mechanisms underlying the pathogenesis of hypertension could lead to the discovery of novel strategies for preventing and treating high blood pressure (BP).

CNS mechanisms driving sympathetic nervous system (SNS) overactivity play an important role in the development and progression of many forms of experimental hypertension (Haspula and Clark, 2018). The brain regions involved in sympathetic regulation include several nuclei located in the brainstem and hypothalamus such as rostral ventrolateral medulla (RVLM), hypothalamic paraventricular nucleus (PVN) and subfornical organ (SFO) (Guyenet, 2006; Dampney, 2016). Accumulating evidence indicates that renin-angiotensin system (RAS) components and inflammatory mediators act on brain nuclei within a central neural network to increase SNS drive that in turn, generates hypertension (Winklewski et al., 2015; Huber et al., 2017). This review presents evidence on how the actions and interactions between components of the brain RAS and CNS immune factors contribute to the pathogenesis of hypertension.

About ten years ago, we discovered that the CNS can be reprogrammed by mild non-pressor challenges that induce a neurological state that is expressed as a sensitized hypertensive response when a challenge (stressor) is presented once again. Thicausal mechanisms in prenatals hypertensive response sensitization (HTRS) is the result of the induction of CNS neuroplasticity in the brain neural pathways controlling the tone of the SNS (Johnson et al., 2015; Johnson and Xue, 2018). Our findings implicate both the brain RAS and CNS inflammatory mechanisms in the neuroplasticity that is responsible for HTRS. This review will focus on the mechanisms of the induction of HTRS produced by the presentation of physiological and dietary challenges presented earlier in the life span in both adults and during the prenatal period.

Besides a prohypertensive axis of the RAS, an antihypertensive axis of the RAS has also been identified and studied extensively regarding its protective role in the development of hypertension (Xu et al., 2011; Medina and Arnold, 2019; Sumners et al., 2020). This review also will discuss briefly recent evidence describing the protective effects of the RAS antihypertensive axis on HTRS.

### The RAS and Proinflammatory Cytokines (PICs) in the CNS

The hypothalamus is a key brain region contributing to the regulation of the cardiovascular system, fluid balance, and energy homeostasis. Maintenance of these functions requires the integration of information from neural and humoral signals initially derived from the systemic (aka the circulating or classic) RAS and the systemic immune system. The SFO and organum vasculosum (OVLT) lie along the lamina terminalis (LT) and the area postrema (AP), located in the caudal medulla, function as sensory circumventricular organs (SCVOs) (Johnson and Gross, 1993). By virtue of lacking a blood-brain barrier, SCVOs can monitor blood-borne humoral factors, including angiotensin (ANG) II, leptin, extracellular osmolality and sodium (Mimee et al., 2013). Information reflecting the levels of blood-borne factors sensed by these structures is then projected through efferent pathways either directly or indirectly to hypothalamic regions including the paraventricular hypothalamic nucleus (PVN), ventromedial hypothalamus (VMH) and the arcuate nucleus (ARC) (Guyenet, 2006). In the hindbrain, there are important cardiovascular nuclei in addition to the AP. These include the solitary tract nucleus (NTS) and RVLM, which have bidirectional connectivity with those hypothalamic nuclei controlling sympathetic activity and BP (Guyenet, 2006; Dampney, 2016).

The RAS plays a fundamental role in the regulation of BP and hydromineral homeostasis. ANG II generated by angiotensin converting enzyme (ACE) 1 acts on ANG type 1 receptors (AT1-R) to produce its major physiological and pathophysiological effects, including direct acting vasoconstriction, baroreflex modulation, sympathetic activation, aldosterone release, triggering oxidative stress, and inflammation (Huber et al., 2017; Miller and Arnold, 2019). Components of the RAS (renin, angiotensinogen, ACE1 and AT1-R) associated with the synthetic cascade generating ANG II comprise a prohypertensive axis of the RAS. There also is a RAS anti-hypertensive axis in which ACE2 cleaves ANG II to form ANG-(1-7) that binds the Mas G protein-coupled receptor (Mas-R) to counter-regulate the effects produced by ANG II/AT1-R interaction. ANG II acting on angiotensin type 2 receptors (AT2-R) also exerts actions that oppose those of ANG II/AT1-R (Xu et al., 2011; Santos et al., 2018; Miller and Arnold, 2019).

The metabolic cascade of the systemic RAS (aka as the circulating or systemic RAS), involves renal renin release to act on renin substrate to generate ANG I and then via ACE1 action, ANG II. Following the elucidation of the components of the classic RAS, comparable elements were identified in many body organs including the brain. The so-called brain RAS is associated with neurons, astrocytes and microglia functions in a paracrine, autocrine, and even intracrine signaling role (Huber et al., 2017; Jackson et al., 2018; Nakagawa et al., 2020). Many CNS nuclei (i.e., the LT, PVN, RVLM, NTS and AP) implicated in cardiovascular control and fluid balance are rich in AT1-R-positive cells and are often innervated by axons containing some components of the RAS (Sumners et al., 2020).

Recent studies demonstrate that either AT2-R or ACE2/ANG-(1-7)/Mas-R are present either within or adjacent to cardiovascular nuclei in the brainstem and hypothalamus that express the AT1-R (Gao and Zucker, 2011; Gao et al., 2012; Sumners et al., 2015, 2020; Medina and Arnold, 2019). Functionally, in contrast to the prohypertensive effects of brain AT1-R activation to increase sympathetic activity and BP, either overexpression or selective activation of central AT2-R decreases BP and plasma norepinephrine levels at least partially by reducing sympathetic outflow (Gao et al., 2008, 2011). Similarly, central infusion of ANG-(1-7) or overexpression of ACE2 has been shown to counteract most typical actions of ANG II or aldosterone and to reduce neurogenic hypertension (Feng et al., 2008, 2010) through decreasing the expression of AT1-R, ACE1 and PICs, and increasing AT2-R and Mas-R expression and nitric oxide production in the PVN (Sriramula et al., 2011).

Recent studies demonstrate that ACE2 is expressed on GABAergic neurons that in turn synapse on excitatory neurons within the hypothalamus (Mukerjee et al., 2019; Xu et al., 2019). ADAM17, an enzyme that catalyzes the cleavage of membrane anchored ACE2, is colocalized with AT1-R. Activation of ADAM17 in glutamatergic neurons leads to a selective increase of sympathetic activity, ultimately contributing to dysautonomia and neurogenic hypertension produced by ANG II or aldosterone (Mukerjee et al., 2019; Xu et al., 2019). These results highlight the importance of both the prohypertensive and antihypertensive axes of the RAS in BP regulation by the CNS.

Systemic treatment with exogenous pyrogen (eg., lipopolysaccharide; LPS) or PIC administration induces enhanced expression of PICs in the brain. Because PICs are large lipophobic polypeptides, they are unlikely to pass the blood-brain barrier. Therefore, as in the case of classic brain RAS communication, the mode of body-brain signaling is worthy of consideration. Current evidence indicates that there are five putative means of informing the brain of systemic inflammation and elevated peripheral PICs. First, sensory circumventricular organs, most notably the OVLT have been implicated in the mediation of fever. There is evidence to indicate that the pyrogenic effect of interleukin (IL)-6 is produced by action on the OVLT to release prostaglandin E_2_, which in turn serves as a humoral mediator to communicate with surrounding tissue in the mode of a volume transmitter (Blatteis et al., 1983; Stitt, 1985; Blatteis, 1990; Harre et al., 2002). Second, there are active transport mechanisms for tumor necrosis factor-α (TNF-α). IL-β and IL-6 from blood into the brain (Banks et al., 1995). Third, brain perivascular cells, which have characteristics of macrophages, are responsive to low systemic doses of LPS and IL-1β. They also release prostaglandin E_2_ to act in a paracrine manner to affect surrounding neural activity (Ericsson et al., 1994; Schiltz and Sawchenko, 2002). Fourth, there is unequivocal evidence showing that PICs, through their binding to receptors on brain endothelial cells, activate a humoral-neural signaling pathway and evoke fever by eliciting prostaglandin E2 synthesis in these cells (Blomqvist and Engblom, 2018). Fifth, visceral afferents arising from the periphery are activated by intraperitoneal administration of LPS or a PIC. Subdiaphragmatic vagotomy abolishes neural activation in the CNS and the central expression of PICs produced by intraperitoneal administration of proinflammatory agents (Maier et al., 1998; Goehler et al., 2000; Dantzer, 2018).

The brain has its own innate immune system, which involves microglia as the resident immune cell. Recent studies indicate that microglia and astrocytes in the brainstem and hypothalamus participate in orchestrating many cardiovascular and metabolic functions (Guyenet, 2006; Dampney, 2016). Glial cell activation involves mobilization of inflammatory signaling pathways involving dynamic changes in the expression and activity of several mediators, such as toll-like receptor 4 (TLR4) and IκB kinase-β/NF-κB, and to release a variety of PICs including TNF-α, IL-6 and IL-1β (De Git and Adan, 2015). Microglial activation and increased PICs in the periphery and CNS have been implicated in the pathophysiology of a variety of cardiometabolic diseases including heart failure, hypertension, and diabetes mellitus (Marina et al., 2016). Recent studies demonstrate that the high BP of the spontaneously hypertensive rat (SHR) and ANG II-induced hypertension are accompanied by microglial activation within the PVN along with elevated PICs (Shi et al., 2010; Shen et al., 2015). The increased levels of blood-borne and central PICs in cardiovascular disease states lead to the neurohumoral activation including sympathoexcitation and upregulation of central RAS activity (Wei et al., 2015, 2018). Blockade of brain microglia or targeted depletion of activated microglia within the PVN attenuates ANG II-induced hypertension, decreases PVN cytokines and reduces cardiac hypertrophy (Shi et al., 2010; Shen et al., 2015). These results support a role for microglial activation and the release of PICs in the pathogenesis of hypertension.

### Interactions of the Brain RAS and PICs in the Pathogenesis of Renin-Angiotensin-Elicited Hypertension

Increased local production of ANG II is one of the main mechanisms responsible for the development and progression of hypertension. Apart from the control of SNS activity and extracellular volume regulation, ANG II also acts as a stimulator of PICs and promotes activation of the brain innate immune system (Shi et al., 2010; Sriramula et al., 2011; Shen et al., 2015). Conversely, both systemic PICs and brain PICs reciprocally interact with the RAS (Winklewski et al., 2015). As previously noted, the body-brain communication of these two systems includes both afferent neural and the humoral pathways over which circulating ANG II and PICs activate the central neural network controlling cardiovascular function (Wei et al., 2013, 2015, 2018). Because either the RAS or inflammatory mediators can contribute individually to the pathogenesis of hypertension, the interaction between RAS components and PICs are likely to have important synergistic effects (Yu et al., 2013; Xue et al., 2016a).

Toll-like receptors are pathogen receptors, which are a major component of the innate immune system (Akira, 2003; Hedayat et al., 2011). A growing body of findings demonstrate that TLR4s modulate inflammatory responses implicated in the development of hypertension. As a prototypic TLR4 ligand, LPS administered acutely activates microglia and increases PICs in the brain and this response is attenuated by blockade of AT1-R (Benicky et al., 2009, 2011). Chronic LPS infusion results in a sustained increase in BP that is accompanied by microglial activation, increased PIC expression and elevated reactive oxygen species (ROS) production in the RVLM (Wu et al., 2012). Francis and colleagues reported that ANG II upregulates TLR4 expression in the PVN. Chronic blockade of brain TLR4 significantly attenuated ANG II-induced hypertension and cardiac hypertrophy. Blockade of TLR4 receptors resulted in reduced ROS generation, myocardial inflammation, NF-κB activity, expression of TNF-α and IL-1β, and altered prohypertensive and antihypertensive RAS components. The blockade resulted in tilting the balance in favor of the antihypertensive response (Dange et al., 2014). SHR have more TLR4s in the PVN, and blockade of these receptors in the PVN prevented the increase in BP and attenuated cardiac hypertrophy by downregulation of inflammatory components and upregulation of anti-inflammatory mediators in the PVN (Dange et al., 2015). These results indicate that in different forms of hypertension, TLR4 is a mediator for microglial activation that further engages the RAS and promotes PIC and ROS production.

NF-κB is a key regulator of PIC expression and the inflammatory response observed in hypertension. In ANG II-induced hypertension, peripheral ANG II infusion increases PICs, ROS, and prohypertensive RAS components (ACE1 and AT1-R) and decreases the antihypertensive components (ACE2 and Mas-R) within the PVN. Bilateral PVN NF-κB blockade decreases the prohypertensive RAS axis and increases the protective antihypertensive RAS axis through an ROS-mediated mechanism. The result is an attenuation of the ANG II-induced hypertensive response (Kang et al., 2009; Cardinale et al., 2012). Furthermore, central pharmacological blockade or genetic knockout of TNF-α has been shown to prevent cardiac hypertrophy and to lower BP in animals with hypertension induced by ANG II through mechanisms similar to that of central blockade of NF-κB (Sriramula et al., 2008, 2013).

Endoplasmic reticulum (ER) stress and autophagy are also implicated in the development of hypertension. In ANG II-induced hypertensive mice there is upregulation of many members of the ER stress pathway in the SFO. Central inhibition of ER stress prevented ANG II-induced hypertension (Young et al., 2012). Likewise, SHRs exhibited greater ER stress and autophagic dysfunction in the RVLM that precedes the development of the hypertensive phenotype, which is dependent on oxidative stress (Chao et al., 2013). Activation of AT1-R increases ER stress and ROS production in the SFO that further activates NF-κB during the development of ANG II-induced hypertension (Young et al., 2015). Collectively, these data suggest that ER stress and autophagy, as well as their interactions with the RAS and PICs in the brain, represent novel cellular and molecular mechanisms underlying the development of neurogenic hypertension.

Recent studies demonstrate that blood-borne PICs induce a pressor response and sympathetic activation through acting on the SFO to increase RAS activity and inflammation. Blockade of AT1-R or ACE or knockdown of AT1-R in the SFO attenuates those PIC-elicited responses (Wei et al., 2013, 2015, 2018; Yu et al., 2018). Tumor necrosis factor-alpha converting enzyme (TACE) generates a soluble form of TNF-α. TACE is upregulated in the SFO and PVN by central infusions of ANG II or IL-1β. The SFO knockdown of the TNF-α receptor 1 or central blockade of TACE ameliorates sympathetic excitation and impaired cardiac hemodynamics in heart failure (Yu et al., 2017, 2019; Korim et al., 2019). These results suggest that the SFO-mediated sympathoexcitatory response to PICs depends on the ambient level of activity of the central RAS and PICs.

Collectively, the studies just described indicate that crosstalk between the RAS and TLR4/NF-κB/TNF-α as well as ER stress and autophagy within the brain tips the balance of the RAS in favor of the prohypertensive axis while decreasing the antihypertensive axis thereby enhancing cardiovascular inflammation. This process results in triggering positive feedback from periphery to brain to drive a progressive increase in sympathetic tone and the pathogenesis of hypertension.

### Brain Inflammation and RAS Activation in Obesity-Induced Hypertension

Obesity is present in epidemic proportions and is a major risk factor for type II diabetes mellitus and hypertension. Either obesity *per se* or consuming a high fat diet (HFD) produces a chronic low-grade inflammatory state characterized by activation of microglia, astrocytes, and increased expression of PICs in the CNS. The mediators of the brain innate immune system, including TLR4, IκB kinase-β/NF-κB (IKKβ/NF-κB), ER stress and autophagy, are involved in obesity-related inflammatory signaling (De Git and Adan, 2015). For example, chronic HFD feeding resulted in increased expression and activity of TLR4 in the hypothalamus (Ropelle et al., 2010; Wang et al., 2012). Eating a HFD for just one day is sufficient to increase hypothalamic IKKβ/NF-κB signaling (Thaler et al., 2012). IKKβ/NF-κB signaling then acts both upstream and downstream from ER stress and autophagy that contributes to the development of leptin resistance and obesity (Zhang et al., 2008; Meng and Cai, 2011). In contrast, brain knockout or central pharmacological inhibition of TLR4 or IKKβ/NF-κB prevents hypothalamic inflammation, improves leptin sensitivity and protects against HFD-induced obesity (Zhang et al., 2008; Kleinridders et al., 2009; Milanski et al., 2009; Meng and Cai, 2011). These results indicate that increased PICs induced by brain innate immune system mediators contribute to the elevated SNS activity and BP in obesity (Hall et al., 2015).

Growing evidence indicates that obesity is associated with overactivation of the RAS in humans and animals (Kalupahana and Moustaid-Moussa, 2012). In addition to its well documented role in BP control, the RAS is involved also in energy homeostasis. Both the classic RAS and adipose tissue RAS play roles in promoting energy storage, whereas in contrast, the brain RAS increases energy expenditure (Grobe et al., 2010; De Kloet et al., 2013). The difference between the peripheral and central RAS effects suggests the presence of a negative feedback mechanism that is responsible for maintaining energy balance (Prior et al., 2010). However, a consequence of the presence of a putative peripheral-central negative feedback pathway for the maintenance of energy balance is that it activates the RAS and PICs to drive increased SNS activity and BP. This may be the crux of the mechanisms underlying the development of obesity-related hypertension. This notion is supported by recent studies showing that although animals with diet-induced obesity (DIO) can restore body weight, insulin levels and leptin sensitivity to normal after being switched to a normal fat diet, BP, renal sympathetic nerve activity (RSNA) and the activity of central RAS and PICs remain high (Prior et al., 2010; Maric et al., 2014). Genetic disruption of AT1-R in leptin-expressing or agouti-related peptide (AGRP)-expressing cells in the ARC abolishes the thermogenic increase in sympathetic nerve activity in response to leptin, HFD, and deoxycorticosterone acetate (DOCA)-salt (Claflin et al., 2017; Deng and Grobe, 2019). In rabbits, three weeks of HFD feeding led to increased BP, heart rate (HR) and RSNA. These are responses mediated by neurogenic mechanisms (Armitage et al., 2012; Lim et al., 2013). Therefore, obesity itself may induce hypothalamic inflammation and sensitization of the brain to circulating sympathoexcitatory factors such as those from the RAS, and these initiate increased BP.

In obese humans and in animal models of DIO, increased SNS activity and BP have also been demonstrated to be associated with increased leptin and activation of a leptin/melanocortin 4 receptor (MC4R) pathway (Hall et al., 2002; Tchernof and Despres, 2013). The CNS structures that mediate leptin’s action include the ARC and PVN and extrahypothalamic regions including the NTS and SFO. Microinjection of leptin into the ARC or PVN increased sympathetic activity (Harlan et al., 2011; Shi et al., 2015), whereas, leptin receptor deletion in the ARC or SFO attenuated the increase in sympathetic activity evoked by leptin (Harlan et al., 2011; Young et al., 2013). In either leptin deficient or leptin receptor deficient mice, the lack of leptin signaling led to attenuated microglial activation and reduced expression of several inflammatory mediators. Since leptin can be considered as a PIC (Lord, 2006), it may be that it is in this role that it produces microglial activation (Gao et al., 2014). De Kloet et al. reported that HFD and obesity activate microglia and astrocytes with a subsequent increase in PICs in the SFO and PVN. This effect was ANG II dependent as some of the responses were reversed by deletion of PVN AT1-R (De Kloet et al., 2014). Other studies have shown that with central inhibition of ACE1, blockade of AT1-Rs or global knockout of AT1-R, there is an abolition of the increase in sympathetic nerve activity to leptin. This suggests that leptin interacts with the brain RAS to regulate sympathetic activity and BP (Hilzendeger et al., 2012; Xue et al., 2016b). Meanwhile, it has been shown that obesity-associated activation of the hypothalamic NF-κB/IKK-β pathway also mediates leptin functions in the CNS (Purkayastha and Cai, 2013). These findings suggest that the interactions and synergism between leptin, the RAS and PICs may be responsible for the obesity-induced increase in sympathetic activity that generates hypertension in DIO.

### Brain RAS Activation and Inflammation in Hypertensive Response Sensitization (HTRS) in Adult Animals

There are many examples of changes in behavioral and physiological responses resulting from prior experience. The impact of exposure to certain stimuli or physiological states can last a lifetime. When a response entails an increase in magnitude because of an antecedent condition, it is considered as an example of sensitization (Kandel et al., 2014). Sensitization involving the CNS has been studied in many conditions but until recently has received no attention in investigation of the pathogenesis of hypertension (Johnson et al., 2015; Johnson and Xue, 2018). In order to study sensitization of the hypertensive response and the role of the CNS in the sensitization process, we developed an Induction-Delay-Expression (IND-DEL-EXP) experimental model. During IND, rats are exposed to various types of challenges that do not produce any sustained increase in BP when administered by themselves. Rats then are allowed a rest period (i.e., DEL) to allow for demonstration of the permanence of the induced state and metabolism of any hormones or drugs that might have been administered. After the DEL, a sensitized hypertensive response to a slow-pressor dose of ANG II was observed in these rats when challenged during a period of EXP. Brains can also be collected at the end of DEL for regional (the LT and PVN) analysis for gene expression of RAS components, PICs and indicators of microglial activation (Johnson et al., 2015; Johnson and Xue, 2018).

We found that peripheral or central pretreatment with non-pressor doses of ANG II, aldosterone, or TNF-α sensitized the hypertensive response to a slow-pressor dose of ANG II. At the end of DEL the mild challenges used for IND upregulated mRNA expression of several components of the RAS and PICs and of microglial marker in the brain, whereas central inhibition of AT1-R, mineralocorticoid receptors, and inflammation reversed the changes in gene expression and also prevented the sensitization of ANG II-elicited hypertension seen during EXP. Importantly, the results showed that either central blockade of the RAS or central blockade of inflammation during IND attenuated both the upregulated expression of RAS components and PICs regardless of the mild challenges used for IND (Xue et al., 2012a, b, 2016a,b). Such findings suggest that a previous encounter with even a mild stressor can chronically upregulate the brain RAS and induce neuroinflammatory cellular and molecular mediators in brain pathways that control sympathetic tone. The result is an increase in neural excitability that is expressed as HTRS when challenged once again with an old or new stressor. The capacity of the RAS components or PICs to mutually up-regulate their expression may be indicative of actions within central sensitization-related positive feed-forward systems which can accelerate the onset and rate of development of hypertension.

In studies of dietary sensitization, 3 weeks of HFD feeding increased white adipose tissue mass, plasma leptin levels and mRNA expression of leptin and leptin receptor in both the LT and PVN (Xue et al., 2016a). Central administration of leptin mimicked HFD sensitization of ANG II-induced hypertension while central leptin antagonist prevented the sensitization (Xue et al., 2016b). Central inhibition of AT1-R, TNF-α synthesis or microglial activation significantly attenuated either HFD- or leptin-induced upregulation of RAS activity, inflammation in the LT and PVN, and HFD or leptin sensitization of ANG II-induced hypertension. These observations indicate that a HFD can predispose rats to display HTRS through leptin-mediated upregulation of RAS components and PICs in the LT and PVN. This leptin-dependent increase in RAS activity and PICs within the CNS may offer a causal link between obesity and hypertension (Xue et al., 2016a, b).

Despite the prohypertensive axis of the RAS playing a predominant role in the sensitization of hypertension, the antihypertensive axis of the RAS has a protective effect against the sensitization process. This is based on its counter-regulatory effects on hypertension. In our studies, low doses of ANG II or aldosterone infusion during IND not only enhances ANG II-induced increases in the expression of renin, AT1-R, and ACE mRNA, but also upregulates mRNA expression of ACE2 and AT2-R in forebrain nuclei (Xue et al., 2012a, b). Furthermore, central ANG-(1-7) normalized the increased mRNA expression of AT1-R and ACE produced by ANG II given during IND while the mRNA expression of ACE2 and Mas-R remained high and abolished the ANG II-induced sensitization in male rats (Xue et al., 2014). The results indicate that the antihypertensive axis of the RAS plays an important counter-regulatory role in protecting against sensitization of ANG II-induced hypertension. The enhanced central ACE2 and AT2-R expression may reflect the activation of inhibitory mechanisms that attempt to buffer against the pressor actions of ANG II and act to keep BP in check (Xue et al., 2012a, b, 2014).

**FIGURE 1:**
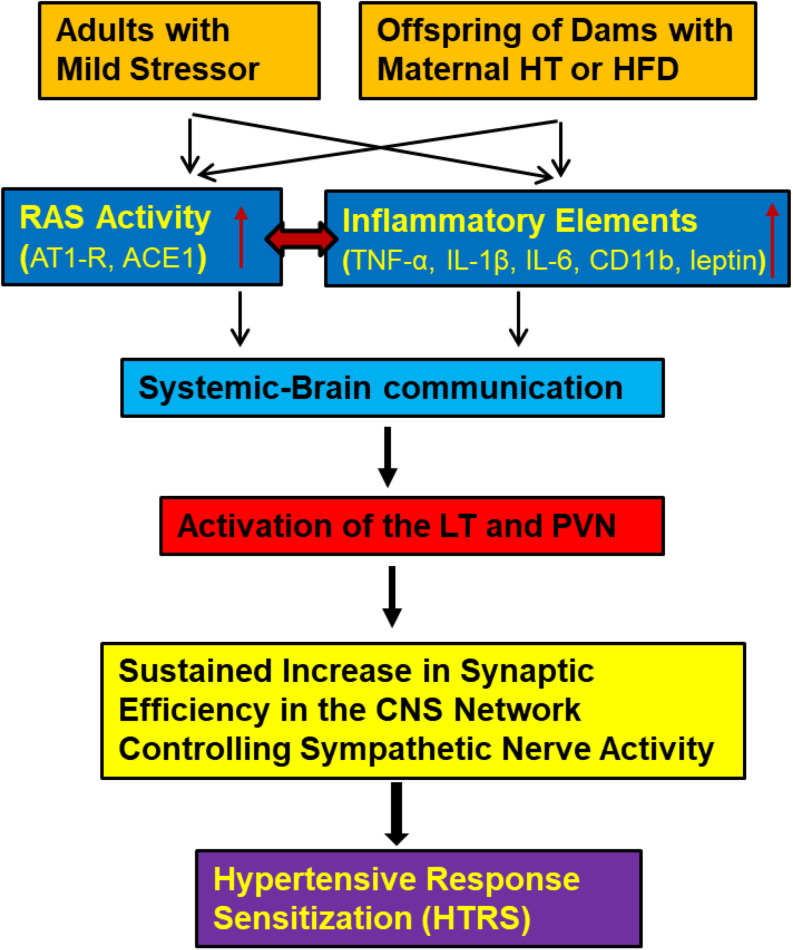
Central cascade of factors mediating hypertensive response sensitization (HTRS). In adult animals challenged with mild physiological or psychological stress or in offspring from dams with maternal hypertension (HT) or high fat diet (HFD) feeding, both the renin-angiotensin system (RAS) and inflammatory elements will be upregulated and act on brain nuclei involved in blood pressure regulation through systemic-brain communication. These central RAS and inflammatory mechanisms will synergistically induce a HTRS. (ACE1, angiotensin converting enzyme 1; AT1-R, angiotensin II type 1 receptor; TNF-α, tumor necrosis factor-α; IL-1β, interleukin-1β; IL-6, interleukin-6; CD11b, cluster of differentiation molecule 11b; LT, the lamina terminalis; PVN, the paraventricular nucleus of hypothalamus).

Taken together, our studies provide insight into the actions of the RAS and of PICs in the hypothalamus to mediate cardiovascular sensitizing effects of mild physiological or metabolic challenges, even though these factors do not induce any apparent immediate abnormalities in the cardiovascular function. The demonstrations of sensitization also indicate that challenges to homeostasis can act to reprogram the neural network controlling BP to generate an enhanced pressor response when a hypertensive stimulus is either sustained or encountered at a point later in life (Johnson et al., 2015; Johnson and Xue, 2018) (Figure 1).

### Brain RAS Activation and Inflammation and Maternal Hypertension- or HFD-Induced Sensitization of Hypertension in Offspring

There is a strong association between maternal health during pregnancy and cardiovascular disease in adult offspring. Many kinds of prenatal insult such as hypertension, obesity or HFD, protein restriction, uteroplacental perfusion insufficiency or glucocorticoid treatment during pregnancy have been demonstrated to have consequences on health of the offspring. Such prenatal challenges alter responses to environmental stressors and predispose human and experimental animals to cardiovascular and metabolic disorders (Reynolds et al., 2013; Alexander et al., 2015).

Increased RSNA and overactivity of the RAS and PICs have been implicated as causal mechanisms in prenatal programming of hypertension (Wu and Berecek, 1993; Lopes et al., 2008; Mizuno et al., 2013, 2014; Washburn et al., 2015; Deng et al., 2016). Either renal denervation (RD) or chronic blockade of the RAS and inflammation reduces elevated BP through restoration of renal and arterial function in offspring of dams after different types of insult to the mother during pregnancy (Wu and Berecek, 1993; Langley-Evans and Jackson, 1995; Intapad et al., 2013; Mansuri et al., 2015; Deng et al., 2016). Recent studies also report that expression of the AT1-R in the SFO and the OVLT was increased in the offspring of protein-restricted dam. Intracerebroventricular injections of an ACE1 inhibitor or an AT1-R antagonist significantly reduced BP in these offspring (Pladys et al., 2004).

The prevalence of obesity in women of childbearing age and during pregnancy has steadily increased over the past two decades (Flegal et al., 2012). Prior et al. demonstrated that the offspring of rabbits fed a HFD had markedly elevated RSNA and pressor responses to central leptin, enhanced sympathetic responses to ghrelin and an exaggerated sympathetic response to acute air-jet stress when compared with offspring from mothers fed a normal fat control diet. This suggests that the higher levels of BP and RSNA may be attributable to changes in central pathways regulating SNS activity (Prior et al., 2014). Also, maternal HFD was associated with hypothalamic inflammation, impaired baroreflex sensitivity and reduced HR variability, implicating abnormalities in autonomic control in the offspring (Samuelsson et al., 2010; Deng et al., 2016; Cesar and Pisani, 2017).

Maternal separation during early life, so called early life stress, sensitizes rats to ANG II-induced hypertension in adult life. Early life stress offspring displayed renal dysfunction, elevated renal and systemic sympathetic activity, enhanced ANG II-mediated vasoconstriction and vascular inflammation and reduced endothelial nitric oxide buffering capacity (Loria et al., 2010, 2011, 2013a,b).

Our studies investigating the effects of gestational maternal challenges on the offspring when they reach adulthood have focused on the role of reprogramming central mechanisms involving the RAS and PICs on SNA. This was done by inducing sensitization of the offspring during the prenatal period by subjecting pregnant dams to ANG II-elicited hypertension or to HFD feeding (Xue et al., 2017; Zhang et al., 2018; Wang et al., 2020). In the offspring of hypertensive dams, we found that the adult male offspring showed upregulated expression of both RAS components and PICs in the LT and PVN and displayed HTRS to a slow-pressor dose of ANG II when compared with the offspring of normotensive dams. Either renal denervation or captopril treatment administered between weaning and adulthood blocked HTRS and reversed the changes in RAS and PIC mRNA expression of the hypertensive dam offspring (Xue et al., 2017). Likewise, in adult offspring of HFD fed dams, upregulated expression of RAS components, NADPH oxidase and PICs were evident in the LT and PVN. These offspring exhibited normal BP at 10 weeks of age, but had blunted cardiac baroreflex function and elevated autonomic sensitivity to central challenges with ANG II or TNF-α. The offspring also showed a significantly greater hypertensive response to a slow-pressor dose of ANG II that was accompanied by an enhanced upregulation of mRNA expression of RAS components, NADPH oxidase and PICs in the LT and PVN (Zhang et al., 2018). These studies further demonstrate that systemic blockade of either the RAS or inflammation in offspring abolished upregulated brain RAS and PICs and sensitization of the hypertensive response to ANG II produced by the pregnant mothers eating a HFD (Wang et al., 2020). These results indicate that maternal HFD feeding during either pregnancy or lactation is sufficient for early life programming of the central HTRS to predispose the next generation to a greater risk of hypertension. The studies also provide a potential therapeutic option to reduce the impact of maternal obesity or HFD on adverse outcomes, especially hypertension, in the offspring.

Sex-specific effects are widely observed in perinatal developmental programming studies and female offspring are protected as compared to male offspring (Dasinger and Alexander, 2016; Colafella and Denton, 2018). We also have found that there are sex differences in sensitization of ANG II hypertension in adult animals and in the offspring of maternal hypertensive mothers, where female animals are protected against the sensitization of hypertension (Xue et al., 2014, 2018).

Taken together, these experiments demonstrate that reprogramming of the mechanisms controlling BP during a sensitive prenatal period produces long-lasting phenotypic changes in the CNS and increased responsiveness to a prohypertensive challenge (Figure 1).

## Summary and Conclusion

In this review, we have described the roles of the RAS and inflammation in the CNS in the development of hypertension. These two systems, either independently or synergistically, activate brain nuclei involved in BP regulation to increase SNS drive that in turn, generates renin-angiotensin-elicited or obesity-related hypertension. We further highlighted the interactions of the RAS and inflammation in the HTRS.

The predisposition of the offspring of mothers exposed to various insults during pregnancy to display HTRS appears to be very similar to sensitization of this response produced by prior challenges that were found in adult animals. The permanence of HTRS is associated with CNS reprogramming instantiated as changes in expression of RAS components and of PICs. The studies on the new models of sensitization provide many possibilities to elucidate central mechanisms underlying the long-term maintenance of sensitization and the etiology of hypertension.

Because maternal hypertension and HFD/obesity are common and rapidly increasing, it is likely that future generations will be at increased risk for cardiovascular diseases including hypertension. Because only half of hypertensive patients show a satisfactory response to conventional treatment (Go et al., 2014; Fryar et al., 2017), it is critical to identify new therapeutic strategies that effectively prevent hypertension or that are useful in treating the disorder. This is especially true for the kinds of high BP that are driven from the CNS. These insights may ultimately reduce the clinical, social and economic burden produced by hypertension.

## Author Contributions

BX conceived, drafted, revised, and approved the manuscript. YZ revised and approved the manuscript. AJ conceived, edited, revised, and approved the manuscript. All authors contributed to the article and approved the submitted version.

## Conflict of Interest

The authors declare that the research was conducted in the absence of any commercial or financial relationships that could be construed as a potential conflict of interest.
